# Gallbladder Empyema and Epidural Abscess Due to *Salmonella* Enteritidis After Treatment of Primary Infection: Case Report and Review of the Literature

**DOI:** 10.1093/ofid/ofad432

**Published:** 2023-08-12

**Authors:** Ambreen Malik, Mamta Sharma, Leonard B Johnson, Ashish Bhargava

**Affiliations:** Division of Infectious Disease, Ascension St John Hospital, Grosse Pointe, Michigan, USA; Division of Infectious Disease, Ascension St John Hospital, Grosse Pointe, Michigan, USA; Division of Infectious Disease, Wayne State University School of Medicine, Detroit, Michigan, USA; Division of Infectious Disease, Ascension St John Hospital, Grosse Pointe, Michigan, USA; Division of Infectious Disease, Wayne State University School of Medicine, Detroit, Michigan, USA; Division of Infectious Disease, Ascension St John Hospital, Grosse Pointe, Michigan, USA; Division of Infectious Disease, Wayne State University School of Medicine, Detroit, Michigan, USA; Division of Infectious Disease, Thomas Mackey Center for Infectious Disease Research, Detroit, Michigan, USA

**Keywords:** nontyphoidal *Salmonella* epidural abscess, nontyphoidal *Salmonella* gallbladder empyema, nontyphoidal salmonellosis, *Salmonella* Enteritidis

## Abstract

Nontyphoidal *Salmonella* can cause gallbladder empyema and disseminated disease in patients with suppressed immune systems. We are reporting a unique case of concomitant gallbladder empyema and epidural abscess due to *Salmonella enterica* subsp *enterica* serovar Enteritidis in a patient who was appropriately treated for the primary *Salmonella* infection complicated by bacteremia. A high degree of suspicion is needed in high-risk patients as timely intervention can avoid life-threatening complications.

Nontyphoidal *Salmonella* (NTS) infections are 1 of the leading causes of foodborne illness worldwide and are estimated to cause approximately 150 million cases of gastroenteritis and 60 000 deaths globally each year [1]. According to the Centers for Disease Control and Prevention (CDC), *Salmonella* causes around 1.35 million infections, with 26 500 hospitalizations and 420 deaths in the United States annually [2]. Salmonellosis is a zoonotic disease associated with consumption of food or water contaminated with animal feces. In immunocompetent individuals, illness is commonly manifested as acute gastroenteritis lasting 4–7 days, and most people recover without treatment. Up to 8% of patients with NTS gastroenteritis develop bacteremia but invasive infection is uncommon, occurring only in 5%–10% of patients who have bacteremia [3]. Risk factors for invasive disease include extreme ages; underlying immunocompromising conditions, particularly human immunodeficiency virus (HIV); malignancy; and hemoglobinopathies. With an underlying suppressed immune system, infection can disseminate to other body sites including bones, joints, skin, heart valves, and, rarely, body spaces and meninges. Gallbladder colonization by NTS is a rare phenomenon as compared to *Salmonella enterica* subsp *enterica* serovar Typhi (*S* Typhi) and has been reported to cause disseminated foci of infection even after resolution of primary infection [4]. Epidural abscess due to NTS is rare, and all the cases that are reported so far had disseminated disease during the episode of primary infection [5]. We present a unique case of gallbladder empyema and epidural abscess due to *Salmonella enterica* subsp *enterica* serovar Enteritidis (*S* Enteritidis) in a patient who was treated for the primary *Salmonella* infection and yet developed disseminated disease.

## CASE REPORT

A 52-year-old Black woman presented after sustaining a fall, which she attributed to bilateral lower extremity (LE) weakness. The patient was unable to stand up and remained on the floor for 3 hours before an ambulance arrived. Approximately 2 days prior to admission, she developed watery diarrhea and associated abdominal pain after eating watermelon and ham that was possibly spoiled. Her past medical history included uncontrolled diabetes mellitus (DM) type 2, hypertension, morbid obesity, and chronic back pain due to remote motor vehicle accident.

Initial vital signs demonstrated tachypnea of 30 breaths/minute, tachycardia of 130 beats/minute, and fever of 38.4°C. Physical examination revealed a power of 4 of 5 in both LEs with no sensory deficits. She had tenderness in the right upper quadrant of the abdomen. She was noted to have urinary retention and thus required placement of a urinary catheter. Her laboratory tests showed normal leukocyte count, mild anemia with hemoglobin of 11 g/dL, acute kidney injury (AKI) with elevation of creatinine to 2.18 mg/dL from baseline of 1 mg/dL, elevated random blood sugar of 349 mg/dL, alanine aminotransferase of 54 IU/L, aspartate aminotransferase of 131 IU/L, and hemoglobin A1c of 12.7%. Alkaline phosphatase and total bilirubin were normal. Ultrasound of the abdomen showed cholelithiasis with changes suggestive of cholecystitis. Both of 2 sets of blood culture grew gram-negative bacilli that were identified as *S* Enteritidis. Ceftriaxone was started. Two days later, her stool culture also grew *S* Enteritidis. Susceptibility results showed that the organism was susceptible to ampicillin, ceftriaxone, and trimethoprim-sulfamethoxazole (TMP-SMX). A transthoracic echocardiogram showed no signs of endocarditis. Magnetic resonance imaging (MRI) of the lumbar spine without contrast showed degenerative disc disease at the L3–L5 level with mild spinal stenosis at the L5–S1 level. Patient was evaluated by neurosurgery; her back pain and LE weakness were attributed to degenerative disc disease with no surgical intervention recommended. Her abdominal pain resolved over the next few days and the patient was discharged to a rehabilitation facility on TMP-SMX for 14 days with a plan to undergo elective cholecystectomy as an outpatient.

**Figure 1.**
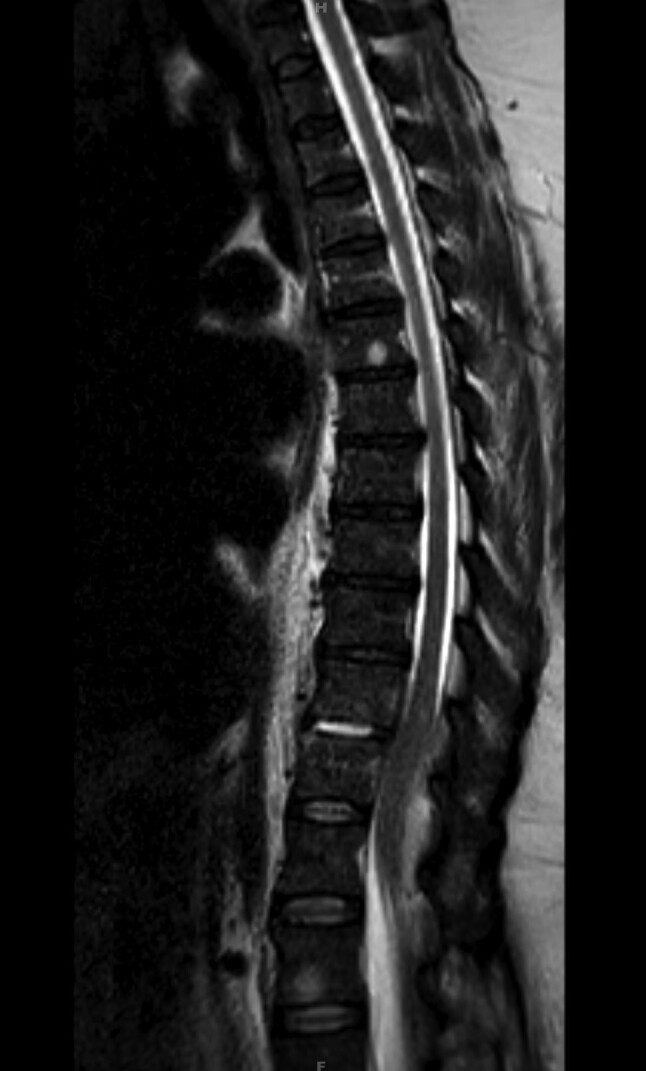
Sagittal view of contrast-enhanced magnetic resonance imaging of thoracic and lumbar spine shows discitis at the T10–T11 disc space level. Abnormal meningeal enhancement is noted at T10–T12, causing some spinal cord compression.

The patient returned 1 month later due to fall and progressive bilateral LE weakness and numbness of the toes. She had abdominal pain but did not have fever. She had minimal improvement in her functional status after discharge from the rehabilitation facility. Neurological examination showed muscle strength of 3 of 5 in both lower extremities with diminished patellar reflex bilaterally. Sensation was intact bilaterally and there was no saddle anesthesia. Tenderness was noted in the right upper quadrant. Laboratory results showed normal leukocyte count, anemia with hemoglobin of 9.9 g/dL, and mild AKI with creatinine of 1.54 mg/dL. Serum bilirubin and liver enzymes were within normal range. An MRI of the thoracic spine with contrast showed discitis of T10–T11 disc space level with epidural fluid collection causing spinal cord compression (Figure 1). Blood cultures showed no growth. The patient underwent thoracic laminectomy with evacuation of the epidural abscess. Based on the recent history of *Salmonella* bacteremia, the patient was started on ceftriaxone. The epidural fluid grew *S* Enteritidis. Patient continued to have right upper quadrant pain and, because of high risk of surgery, underwent cholecystostomy with drainage of purulent bile, which also grew *S* Enteritidis. Susceptibility results from bile and epidural fluid showed that the *Salmonella* was susceptible to ampicillin, TMP-SMX, fluoroquinolones, and ceftriaxone. Later, she underwent cholecystectomy and was found to have gallbladder empyema with extensive necrosis. She recovered from surgeries well and did not have residual neurological deficit. The patient improved clinically and was discharged on ceftriaxone 2 g daily for 6 weeks. She was doing well at 1-week follow-up after discharge. Antibiotics were extended for a total of 12 weeks based on her elevated inflammatory markers. At the end of the course, she did not have any neurological deficit.

## DISCUSSION AND REVIEW OF THE LITERATURE

Salmonellae are gram-negative, non-spore-forming, facultative anaerobic bacilli belonging to the Enterobacteriaceae family and subdivided into 2 species: *Salmonella bongori* and *Salmonella enterica*. Among these 2 species, >2500 serovars have been reported based on antigenic diversity of somatic O antigens (component of lipopolysaccharide) and flagellar (H) antigens. Almost all of the serotypes pathogenic for humans belong to *S enterica* species. NTS infection is caused by *Salmonella* species other than *S* Typhi and *S enterica* subsp *enterica* serovar Paratyphi. The incidence of NTS infections is highest in summers in temperate climates and rainy seasons in tropical areas.

NTS can be acquired from multiple animal reservoirs, and transmission to humans can occur by ingestion of contaminated food of animal origin, especially eggs, poultry, undercooked meat, dairy products, and fresh produce contaminated with manure. Human or animal waste can contaminate the surface of fruits and vegetables and, if not removed by washing, can be introduced into the food. In 2019, the CDC reported a multistate foodborne outbreak caused by *Salmonella* associated with precut melons that infected 137 people from 10 states [6]. Once the bacteria are ingested with contaminated food or water, it has the ability to display an adaptive acid tolerance, which facilitates its survival in the stomach and passage to the small intestines. *Salmonella* invades the intestinal epithelial cells by bacteria-mediated endocytosis with the help of an encoded protein called type III secreting system within *Salmonella* pathogenicity island 1 (SPI-1 T3SS) [7]. NTS also induces a secretory response in the intestinal epithelium initiating recruitment and transmigration of neutrophils into the intestinal lumen causing intestinal inflammation, which contributes to fluid secretion and diarrhea through disrupted epithelial barrier. Studies have demonstrated that *Salmonella* also uses SPI-1 T3SS to downregulate the host inflammatory response to intestinal invasion, contributing to asymptomatic colonization of the intestines typically seen with NTS infections. Bacteria can enter the biliary tract via portal vein bloodstream, directly from small intestines via the duodenal papilla or direct transfer into the gallbladder from the liver. *Salmonella* Typhi is known to colonize gallbladder due to its ability to form biofilms on the gallstones and cholesterol-coated surfaces as demonstrated by Crawford et al [8]. It is proposed that nontyphoidal strains can also form gallstone biofilm in similar fashion, leading to chronic carriage in the gallbladder, but this is infrequent and reported in only 0.2%–0.6% of adults infected by NTS as compared to *S* Typhi, which causes chronic carriage in 1%–4% of patients [9]. Immunosuppressive conditions and cholelithiasis are considered predisposing risk factors for gallbladder colonization [10].

The biliary tract including liver and gallbladder are the most common extraintestinal sites of *Salmonella* infection. Although acute cholecystitis is a well-reported complication of infection caused by *S* Typhi, it is rarely seen with NTS and only 10 cases of acute cholecystitis are reported in literature. Empyema of gallbladder is a much rarer complication. In the literature, only 4 cases of empyema of gallbladder due to NTS are reported and only 1 of them had invasive infection after treatment of primary infection (Table 1). That patient had underlying chronic granulomatous disease and was initially treated with 10 days of intravenous ceftriaxone for gastroenteritis and bacteremia due to *S* Enteritidis. After initial improvement, she relapsed and presented after 2 weeks with gallbladder empyema and did well after laparoscopic cholecystectomy [4].

**Table 1.** Summary of Published Cases of Empyema of the Gallbladder Caused by *Salmonella* Enteritidis

Study, Year [Reference]	Age, y	Sex	Presenting Symptoms	Risk Factors	Source of Positive Culture	*Salmonella* Species	Surgical Intervention	Antibiotics	Outcome
Yamashita et al, 2018 [4]	40	F	Fever, abdominal pain, diarrhea	CGD	Blood, stool, bile	*S* Enteritidis	PTC, cholecystectomy	Levofloxacin, ceftriaxone	R
Lianos et al, 2019 [11]	32	M	Fever, abdominal pain, vomiting, diarrhea	None	Stool, bile	Not reported	Cholecystectomy	CRO, ciprofloxacin	R
Juma et al, 2019 [12]	41	M	Fever, abdominal pain, vomiting, diarrhea	Travel to Guinea	Bile	*S enterica* subsp *salamae*	PTC, cholecystectomy	Piperacillin-tazobactam, CRO, ciprofloxacin	R
Zhao et al, 2020 [13]	90	M	Fever, abdominal pain, vomiting, diarrhea	Eating oysters	Blood, stool, bile	*Salmonella* group D	Cholecystectomy	CRO, meropenem	R
Present study	52	F	Leg weakness, abdominal pain, fever, diarrhea	Uncontrolled DM, morbid obesity	Blood, stool, bile, epidural abscess	*S* Enteritidis	Cholecystectomy, drainage of epidural abscess	CRO, TMP-SMX	R

Abbreviations: CGD, chronic granulomatous disease; CRO, ceftriaxone; DM, diabetes mellitus; F, female; M, male; PTC, percutaneous transhepatic cholecystostomy; R, recovered; TMP-SMX, trimethoprim-sulfamethoxazole.

Epidural abscess is another rare clinical manifestation of NTS infection, with only 16 cases reported in the literature (Table 2). Sickle cell disease, connective tissue disease, HIV infection, poorly controlled DM, and myelofibrosis were reported as underlying risk factors for disseminated disease. The most common location identified was the lumbar spine followed by thoracic and cervical spine. *Salmonella* Enteritidis was the most common species identified. Commonly used antibiotics were third-generation cephalosporins, ciprofloxacin, penicillin, and TMP-SMX. Seven patients required surgical intervention in addition to the antibiotic.

**Table 2.** Summary of Published Cases of Epidural Abscess Caused by Nontyphoidal *Salmonella*

Study, Year [Reference]	Age, y	Sex	Presenting Symptoms	Risk Factors	Source of Positive Culture	Location	*Salmonella* Species	Surgical Intervention	Antibiotics	Other Foci of Infection
Gardner, 1985 [14]	2	M	Fever, irritability	SCD	Blood, epidural fluid, CSF	L3	*S* Enteritidis	Laminectomy and evacuation of epidural abscess	Ampicillin (4 wk) and chloramphenicol (3 wk)	Meningitis, lumbar, and rib osteomyelitis
Akagi et al, 1998 [15]	53	M	Diarrhea, paresthesia, weakness of bilateral upper and lower extremities	Alcohol abuse	Stool, epidural tissue	C5–C7	*S* Enteritidis	Decompression and anterior cervical fusion with bone graft	Cefmetazole and amikacin	None
Diebold et al, 2003 [16]	5	M	Fever, diarrhea, headache	SCD	Blood, stool, frontal subcutaneous fluid	Frontal (Intracranial)	*S* Enteritidis	Frontal bone resection and drainage of epidural abscess followed by reconstruction	Amoxicillin-clavulanic acid (2 wk) followed by CRO (2 wk) and then amoxicillin-clavulanic acid (2 wk)	Frontal osteitis, Frontal skin abscess
Ozturk et al, 2006 [17]	53	F	Leg pain	SLE	Epidural tissue and fluid, trochanteric abscess	T8–T10	*S* Typhimurium	Hemilaminectomy, drainage of abscess	Ciprofloxacin	Trochanteric abscess
Blázquez et al, 2009 [18]	2	F	Fever, diarrhea, vomiting	Brain surgery, dexamethasone use	Epidural fluid, CSF, stool	Parietal (intracranial)	*S* Enteritidis	Not done	Ceftazidime (1 wk), chloramphenicol (4 wk), cefotaxime (2 wk)	Brain abscess
Hachfi et al, 2009 [19]	37	M	Fever, headache	HIV	Frontal epidural fluid	Intracranial	*S* Typhimurium	Craniotomy and evacuation of abscess	Ciprofloxacin (12 wk)	Frontal osteomyelitis
Choi et al, 2010 [20]	62	M	Back pain	None	Epidural fluid	L3	*Salmonella* group D	None	CRO, ciprofloxacin	None
de Araujo et al, 2012 [21]	69	F	Back pain, fever	SLE	Blood, epidural fluid	T6–T7	*S* Enteritidis	Evacuation of abscess	Cefepime	None
Oki et al, 2016 [22]	54	M	Fever, diarrhea, back pain	Alcohol abuse	Blood, stool, CSF	L4–L5	*S* Enteritidis	None	CRO, ciprofloxacin	Meningitis
Fareed et al, 2017 [23]	50	M	Fever, diarrhea, back pain	Myelofibrosis	Blood	L4–L5	*Salmonella* group D	Laminectomy, drainage of epidural abscess	CRO (8 wk)	None
Dahlberg et al, 2018 [24]	54	M	Back pain, LE numbness and weakness	None	Epidural tissue and fluid	L5–S1	*S* Agbeni	Decompression laminectomy, evacuation of epidural abscess	CRO	None
Majid et al, 2018 [25]	54	F	Back pain, LE numbness and weakness	None	Blood, epidural fluid, urine	T12–L3	*S* Enteritidis	Hemilaminectomy and drainage of epidural abscess	CRO, ciprofloxacin	Endocarditis, UTI
Hirai et al, 2020 [5]	52	M	Fever, back pain	Well-controlled DM	Blood, stool, epidural fluid	L1–L5	*S* Altona	Drainage of epidural abscess	CRO, ciprofloxacin	Psoas abscess, spinal osteomyelitis
Elnour et al, 2022 [26]	27	M	Fever, back pain	SCD, splenectomy	Blood, sputum, epidural fluid	T4–T7	*Salmonella* (non- Typhi)	Decompression, posterior T4–T7 laminectomy, and drainage of epidural abscess	CRO (4 wk) followed by TMP-SMX (2 wk)	Pneumonia, discitis
Hsu et al, 2022 [27]	85	F	Fever, back pain	DM	Blood, urine	T12–S1	*Salmonella* group C	Surgical debridement, decompression, and drainage of epidural abscess	Doripenem (8 wk) followed by colistin (5 wk)	Discitis
Daggett et al, 2022 [28]	1	M	Fever, diarrhea, vomiting, difficulty ambulating	Travel to Florida	Surgical tissue	S1–S2	*S* Enteritidis	Hemilaminectomy and evacuation of epidural abscess	CRO (6 wk) followed by amoxicillin (3 mo)	Spinal osteomyelitis
Present study	52	F	Leg weakness, abdominal pain, fever, diarrhea	Uncontrolled DM	Blood, stool, bile, epidural fluid	T10–T12	*S* Enteritidis	Cholecystectomy, drainage of epidural abscess	CRO, TMP-SMX	Gallbladder empyema

Abbreviations: CSF, cerebrospinal fluid; CRO, ceftriaxone; DM, diabetes mellitus; F, female; HIV, human immunodeficiency virus; LE, lower extremity; M, male; SCD, sickle cell disease; SLE, systemic lupus erythematosus; TMP-SMX, trimethoprim-sulfamethoxazole; UTI, urinary tract infection.

In our literature review, we have found only 1 patient who developed epidural abscess and gallbladder empyema due to *S* Enteritidis after treatment of primary infection and, to our knowledge, this is the first case reported. We can postulate here that cholelithiasis and poorly controlled DM might have contributed to colonization of gallbladder in our patient. The reason for developing invasive disease despite treatment of primary infections could be explained by the fact that antibiotics might not be effective in eliminating carriage state and instead prolong the duration of carriage state. Also, poorly controlled DM and morbid obesity could have contributed toward invasive NTS disease as these factors are known to cause immunosuppression. The definitive study to diagnose epidural abscess is contrast-enhanced MRI, which was delayed in this case due to AKI. In our literature review, DM was reported in 2 cases as the underlying risk factor for invasive NTS disease, whereas morbid obesity has not been reported as a risk factor for invasive NTS disease.

Uncomplicated NTS gastroenteritis is a self-limiting disease, and antimicrobials should not be routinely used as they may not eliminate the bacteria and may select for resistant strains. Antimicrobial therapy should be considered in certain populations who are at increased risk of developing invasive infections and include neonates; patients aged >50 years; and patients with underlying immunosuppressed condition, cardiac valvular or endovascular abnormalities, or prosthetic joints. Due to increasing reported resistance, patients with bacteremia should be empirically treated with third-generation cephalosporins or fluoroquinolones unless the susceptibilities are known. Recommended antimicrobial therapy for epidural abscess is intravenous ceftriaxone or ampicillin for 6 weeks in addition to source control if indicated.

Treatment of patients with asymptomatic carriage of NTS is controversial; a randomized, placebo-controlled trial conducted on food workers in Thailand using two 5-day regimens (norfloxacin 500 mg twice daily and azithromycin 500 mg daily) showed no benefits among the 2 groups but instead increased the risk of reinfection and antimicrobial resistance [29].

We hypothesize, based on our case and review of the literature, about the potential for colonization of NTS among patients with diseased gallbladder and an immunocompromised state that can put them at risk for invasive disease even after adequate treatment [10]. Therefore we suggest careful monitoring and follow-up for these at-risk patients with NTS bacteremia.

## CONCLUSIONS

NTS can cause colonization of the gallbladder in patients with immunocompromised conditions and cholelithiasis. These patients can develop invasive NTS disease even after the adequate treatment of primary infection as antibiotics are not effective in eliminating the colonization state. Careful monitoring and follow-up should be considered for these at-risk patients with NTS bacteremia. Prompt diagnosis, treatment, and early interventions for invasive NTS disease are important to prevent life-threatening complications.

## Notes

***Patient consent.*** Written informed consent was obtained from the patient. The design of the study conforms to the standards currently applied in the country.
